# Dissecting the difference between positive and negative brain health sentiment using X data

**DOI:** 10.3389/fdgth.2025.1648671

**Published:** 2025-11-26

**Authors:** Piotr Religa, Michel-Edwar Mickael, Marzena Łazarczyk, Norwin Kubick, Ibrahim F. Rehan, Jarosław Olav Horbańczuk, Asmaa Elnagar, Mariusz Sacharczuk, Atanas G. Atanasov

**Affiliations:** 1 Department of Medicine, Karolinska Institute, Solna, Sweden; 2 Institute of Genetics and Animal Biotechnology of the Polish Academy of Sciences, Jastrzebiec Magdalenka, Poland; 3 Department of Biology, Institute of Plant Science and Microbiology, University of Hamburg, Hamburg, Germany; 4 Ludwig Boltzmann Institute of Digital Health and Patient Safety, Medical University of Vienna, Vienna, Austria

**Keywords:** sentiment analysis, brain health, social media, coping strategies, cognitive behavioral therapy, medication and diet

## Abstract

**Introduction:**

Human behavior is significantly influenced by emotions, with negative sentiments such as fear and anxiety driving various coping mechanisms, including cognitive behavioral therapy (CBT), dietary changes, and medication use. Social media platforms like X (formerly Twitter) offer valuable insights into these behaviors due to their real-time, user-generated content. While previous research has explored general sentiment on X (formerly Twitter), there has been limited focus on the reasons behind negative sentiments and the coping strategies employed, particularly in relation to brain health.

**Methods:**

We analyzed 390,000 X-posts tagged with #brain and #health, categorizing them into positive, negative, and neutral sentiments. We then investigate the use of CBT techniques, dietary adjustments, and specific medications across these sentiments.

**Results:**

Our findings reveal distinct patterns in how negative and positive sentiments are expressed and managed on social media. Negative sentiments are often linked to serious health concerns, such as COVID-19 and brain inflammation, and exhibit various cognitive distortions. These X-posts also mention coping strategies like using medications such as lorazepam and simvastatin, or consuming comfort foods like pizza. In contrast, positive sentiments emphasize resilience and improvement, with mentions of mindfulness, supplements, and medications like doxycycline and pregabalin. The study also highlights the risk of disseminating information about dietary and drug supplements that may not be suitable for public use due to serious side effects, such as Chaga mushrooms, which, despite being associated with positive sentiment, are known to cause renal failure in certain cases.

**Conclusion:**

Overall, the study profiles the use of positive and negative brain health sentiment of X, which underscores both the advantages and risks of using X (formerly Twitter) as a platform for sharing brain health-related information.

## Introduction

Human behavior is often influenced by various emotions, with negative sentiments playing a compelling role (1). Negative emotions such as fear, anxiety, and frustration can be triggered by various life events, health challenges, and social circumstances (2, 3). These emotions often drive behaviors aimed at coping with or mitigating a perceived threat or discomfort. Coping mechanisms can vary widely, from adopting therapeutic practices like Cognitive Behavioral Therapy (CBT) to seeking comfort in familiar foods, using supplements, or even self-medicating (4–6). Understanding the behavioral responses to negative sentiment is crucial, as they significantly impact mental health and well-being (7).

Social media platforms, particularly X (formerly Twitter), have become invaluable resources for analyzing human behavior on a large scale (8). X's real-time nature and widespread use make it an ideal platform for capturing immediate reactions and sentiments to various events, including those related to health (9). The vast amount of user-generated content on X allows researchers to observe patterns of emotion, behavior, and interaction that offer insights into how people collectively respond to health-related issues (10). Through sentiment analysis, researchers have classified X-posts into positive, negative, or neutral categories, thereby gaining a deeper understanding of the public's emotional landscape (11).

Previous studies have utilized X to analyze positive and negative sentiments, particularly in the context of mental health issues such as depression and anxiety (9, 12–15). These studies have highlighted that while positive sentiments are more common, negative sentiments tend to elicit stronger engagement, such as reposts and comments (16). However, there has been limited exploration into the underlying motives behind these negative sentiments, and the coping mechanisms people employ to manage their emotions, especially in relation to brain health. Understanding these aspects is critical for developing interventions that can better support individuals dealing with negative emotions and health concerns.

In our study, we addressed these gaps by analyzing X-posts mentioning “brain” and “health”, categorizing them into positive, negative, and neutral sentiments. We conducted several analyses to investigate the reasons behind negative sentiments and how people cope with these emotions. Specifically, we examined the use of therapeutic approaches like CBT, dietary changes, and the consumption of drug supplements and medications. By exploring these coping strategies, we aimed to provide a comprehensive understanding of how individuals deal with health-related challenges on social media, offering new insights into the intersection of sentiment, behavior, and health management.

## Methods

### Data collection

Data extraction from X was conducted using the third-party software Fedica Analytics Platform (https://fedica.com/), which served as the main tool for this process. Fedica's functionalities enabled the extraction of various data types, including X-posts, posts, likes, and mentions, offering a comprehensive overview of activity on X. X-POSTS shared between 6/1/2023 12:00:00 PM and 12/1/2023 12:00:00 PM (1 of June 2023- 1 of December 2023) that simultaneously contained the terms “brain” and “health” were analyzed with Fedica (https://fedica.com/) on 12/12/2023 (12 of December 2023) (17).

### Data analyses

Data was analyzed in Python 3.0. First data was collected from Fedica. After initial analysis of the number of posts and reposts, we developed a function to clean text by removing punctuation and numbers, applied it to the dataset, and created a new DataFrame with the cleaned posts and repost counts, simplifying the data by eliminating the original dataset and intermediate steps.

### Sentiment analyses

We performed sentiment analysis on the X-posts in the updated dataset using TextBlob, categorizing them into positive, negative, and neutral sentiments based on their polarity scores (18, 19). We counted the number of X-posts for each sentiment and tracked their indices in separate lists. We then calculated the total number of reposts corresponding to each sentiment category—negative, positive, and neutral—by summing the repost counts from the updated dataset. The sentiment and repost data were then visualized using bar charts to compare the number of X-posts and reposts by sentiment category. Additionally, we calculated and plotted the ratio of reposts to X-posts for each sentiment, providing a clear comparison of how different sentiment types of influence reposting behavior. The visualizations highlighted key trends in sentiment-driven engagement on the platform.

### Statistical analyses

To examine associations between categorical variables in the dataset, we used chi-square tests of independence. For each analysis, a contingency table was constructed with rows representing one categorical variable and columns representing another (e.g., sentiment type, post type, or word category). The chi-square test evaluates whether the observed distribution of counts differs significantly from what would be expected under independence. Statistical significance was assessed at *p* < 0.05. All analyses were performed using Python 3.11 with the SciPy and Pandas libraries.

### Exploration of sentiment causes

We explored the nature of emotions and topics driving negative sentiment through various analyses. First, we calculated the total occurrences and normalized sentiment ratios (negative, positive, and neutral) for emotions like Depression, Fearful, Hate, and Annoyed. Secondly, we utilized the WordCloud library to visually represent the most common words associated with negative and positive sentiments in our dataset (20). We first extracted and combined the text from all posts classified as negative into a single string. Using this text, we generated a word cloud with a light blue background to highlight the frequency of words related to negative sentiments. Similarly, we created a word cloud for positive sentiment posts, using a light green background. Each word cloud was configured to display up to 5,000 words, with additional contouring for visual clarity. Thirdly, we applied the LDA (Latent Dirichlet Allocation) model to identify the four most prominent topics within the negative sentiment X-posts (21, 22). LDA is a generative statistical model used for discovering abstract topics in a collection of documents by analyzing word co-occurrence patterns. This technique helps in uncovering the underlying themes or topics by grouping similar words and phrases, allowing us to understand the prevalent themes associated with negative sentiments in the dataset.

### Linguistic analysis of speech acts

We analyzed X-posts categorized by sentiment—positive, negative, and neutral—to classify them according to their speech acts (23). We first extracted X-posts corresponding to each sentiment category from the dataset. Using a classification function, we categorized each tweet as a “Question,” “Request,” or “Assertion” (23, 24). We then maintained separate counters for each type of speech act within each sentiment category. After processing all X-posts, we aggregated and reported the counts of each speech act type for positive, negative, and neutral sentiments, providing insights into the distribution and nature of speech acts across different sentiment categories.

### Cognitive distortion analyses

We first analyzed complaint-related language within negative sentiment X-posts by identifying and counting specific words associated with complaints. We calculated the ratio of complaint words to the total number of words in these X-posts to quantify the prevalence of complaints. Following this, we repeated the analysis for various cognitive distortions, including categories such as Fallacy of Change, Blaming, Emotional Reasoning, and others, by examining the occurrence of specific distortion-related terms (25). We then compared the ratios of these cognitive distortions across negative, positive, and neutral sentiment X-posts. Finally, we visualized the ratio of emotional reasoning words in a pie chart to illustrate the distribution of these terms across different sentiment categories.

### Cognitive load analyses

In this section, we evaluated the readability of X-posts categorized by sentiment—negative, positive, and neutral—using the Flesch-Kincaid Grade Level score (26, 27). We first combined the X-posts from each sentiment category into a single DataFrame for analysis. We then calculated the Flesch-Kincaid Grade Level for each tweet, which provides an estimate of the U.S. school grade level required to understand the text (28). This score was computed using the flesch_kincaid_grade function from the textstat library. The calculated readability scores were then aggregated by sentiment category to determine the average readability across negative, positive, and neutral X-posts. All metrics were evaluated for differences across sentiment categories.

Normality of the cognitive load metrics was assessed using the Shapiro–Wilk test. All metrics significantly deviated from a normal distribution (*p* < 0.001), leading to the use of non-parametric statistical tests for group comparisons. Given the non-normal distribution of metrics (Shapiro–Wilk test, *p* < 0.001 for all), non-parametric tests were employed: Kruskal–Wallis *H*-test to determine if at least one sentiment group differed significantly for each metric. *post-hoc* pairwise comparisons using Dunn's test with Bonferroni correction to identify specific group differences. All analyses were performed in Python (v3.12) using the scipy and scikit-posthocs libraries. Effect sizes were not reported due to the large sample size, but extremely small *p*-values (*p* < 0.001) indicated strong statistical differences between sentiment groups.

### Prevalence of therapeutic discourse

In this analysis, we aimed to examine the prevalence of therapeutic discourse within X-posts categorized by sentiment: negative, positive, and neutral. We identified specific keywords related to four therapeutic discourse categories—Self-Compassion, Resilience, Coping Mechanisms, and Therapeutic Support—and calculated the percentage of X-posts in each sentiment category that mention these keywords. By applying this analysis, we sought to understand how often X-posts reflect themes of self-compassion, resilience, and coping strategies across different sentiment categories.

### Prevalence of cognitive-behavioral therapy (CBT)

Here, we quantified the presence of cognitive-behavioral therapy (CBT) techniques, specifically reframing and Emotion Regulation Strategies within X-posts categorized by sentiment (29). We first identified X-posts containing keywords related to reframing by applying a function that checks for these keywords. Using this approach, we calculated the absolute counts of X-posts containing reframing keywords across three sentiment categories: negative, positive, and neutral.

### Prevalence of food, drugs, and drug supplements

The analysis explores the prevalence of specific themes—food types, stress management techniques, drugs, and dietary supplements—within X-posts categorized by sentiment (negative, positive, and neutral) using key libraries and APIs. It first identifies and categorizes mentions of various food types in X-posts, visualizing the relationships between sentiments and food mentions through a network graph to show associations and clustering based on co-occurrence. X-posts discussing stress management are filtered by specific keywords to examine how often different foods are mentioned in the context of stress management across sentiment categories. Drug mentions are identified using the FDA's openFDA API, allowing for comparison of drug prevalence across sentiments to provide insights into sentiment associations with different drugs. Finally, mentions of dietary supplements are counted and compared across sentiment categories, revealing trends in supplement-related discussions within X-posts. A Chi-square test of independence was applied to test whether the distribution of food types differed significantly between negative, neutral, and positive sentiment posts.

### Ethical aspects

This research uses publicly available, anonymized data in full compliance with the terms, conditions, and privacy policies of X/Fedica. Ethical approval was unnecessary as the study did not involve any proactive intervention and was conducted solely with existing public data. All data were anonymized, ensuring that no identities or personal information linked to specific X user accounts are disclosed in this study.

## Results

**Figure 1:**
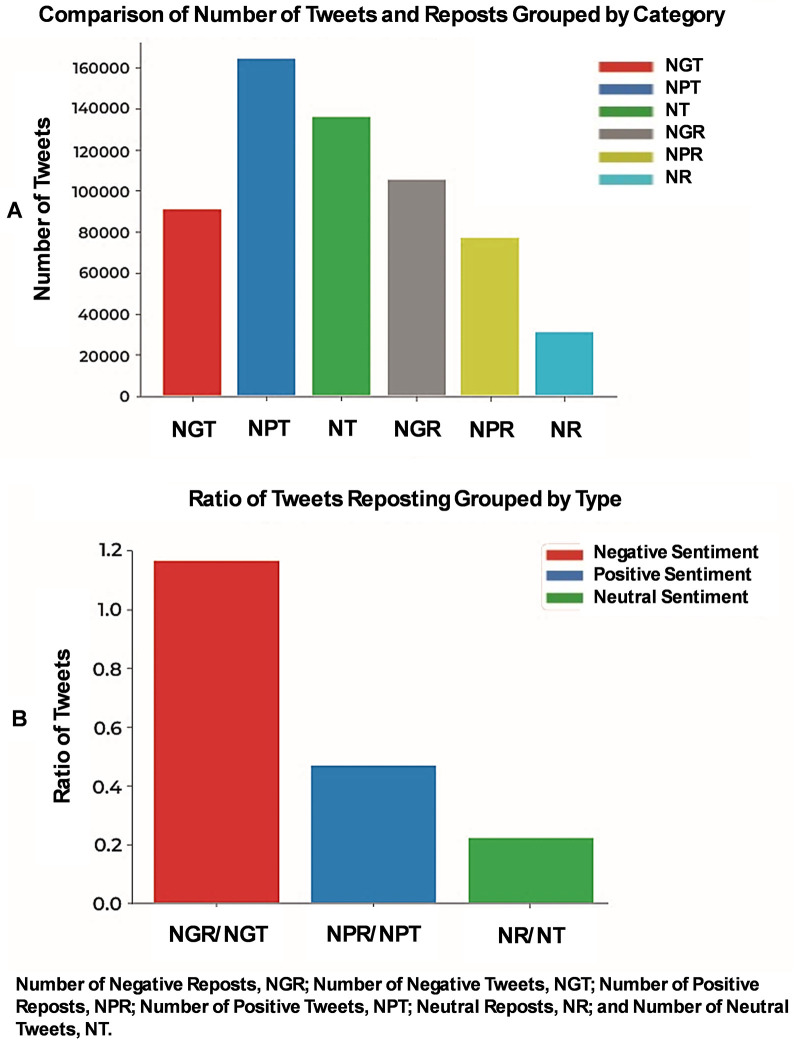
Prevalence and reposting trends of health-related X-posts. The collected data were categorized based on sentiment into negative, positive, and neutral categories using TextBlob, as highlighted in the Methods section. **(A)** Our results indicate that the positive sentiment category had the highest number of X-posts. **(B)** Interestingly, the ratio of reposts to posts shows that negative sentiment X-posts are reposted more frequently than those in other categories.

By analyzing approximately 390,000 X-posts categorized by sentiment, we found around 90,000 negative, 160,000 positive, and 138,000 neutral posts. Interestingly, negative sentiment posts were reposted more frequently than positive or neutral ones (Figure 1A). The ratio of reposts to posts was 1.18 for negative sentiment and 0.44 for positive sentiment (Figure 1B). A chi-square test of independence confirmed that reposting behavior was significantly associated with sentiment (*χ*^2^ = 58,357.92, df = 2, *p* < 0.0001), with negative posts being reposted disproportionately more than expected, while positive and neutral posts were reposted less frequently.

**Figure 2:**
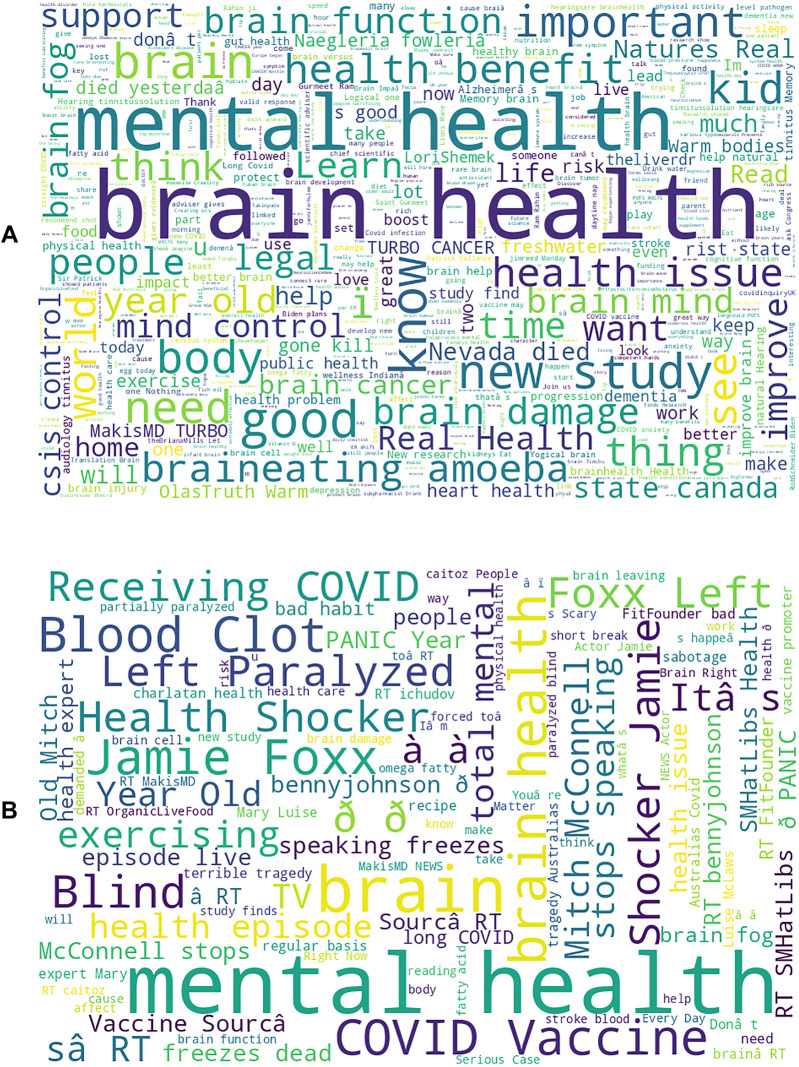
Perception of current events differs based on sentiment. **(A)** Individuals with positive sentiments tend to view negative events as opportunities to “learn”, “improve”, and “help” These X-posts often reflect efforts to remain resilient in the face of adversity, employing various adaptive strategies such as “mind control” **(B)** In contrast, negative sentiment is more focused on the negative aspects of celebrity news and current events, with these X-posts tending to report and analyze such events more extensively than their positive sentiment counterparts.

Although both negative and positive sentiment tweets share a common theme of concern for mental and brain health, they differ in their approach. While Positive sentiment X-posts often include words associated with positive “improve” and “learn”, negative sentiment X-posts tend to reflect a more pessimistic view of health news, using phrases like “left paralyzed”, “health shocker”, and “COVID”. These posts also frequently highlight negative life events affecting public figures, such as Mitch McConnell and Jamie Foxx (Figures 2A,B).

**Figure 3:**
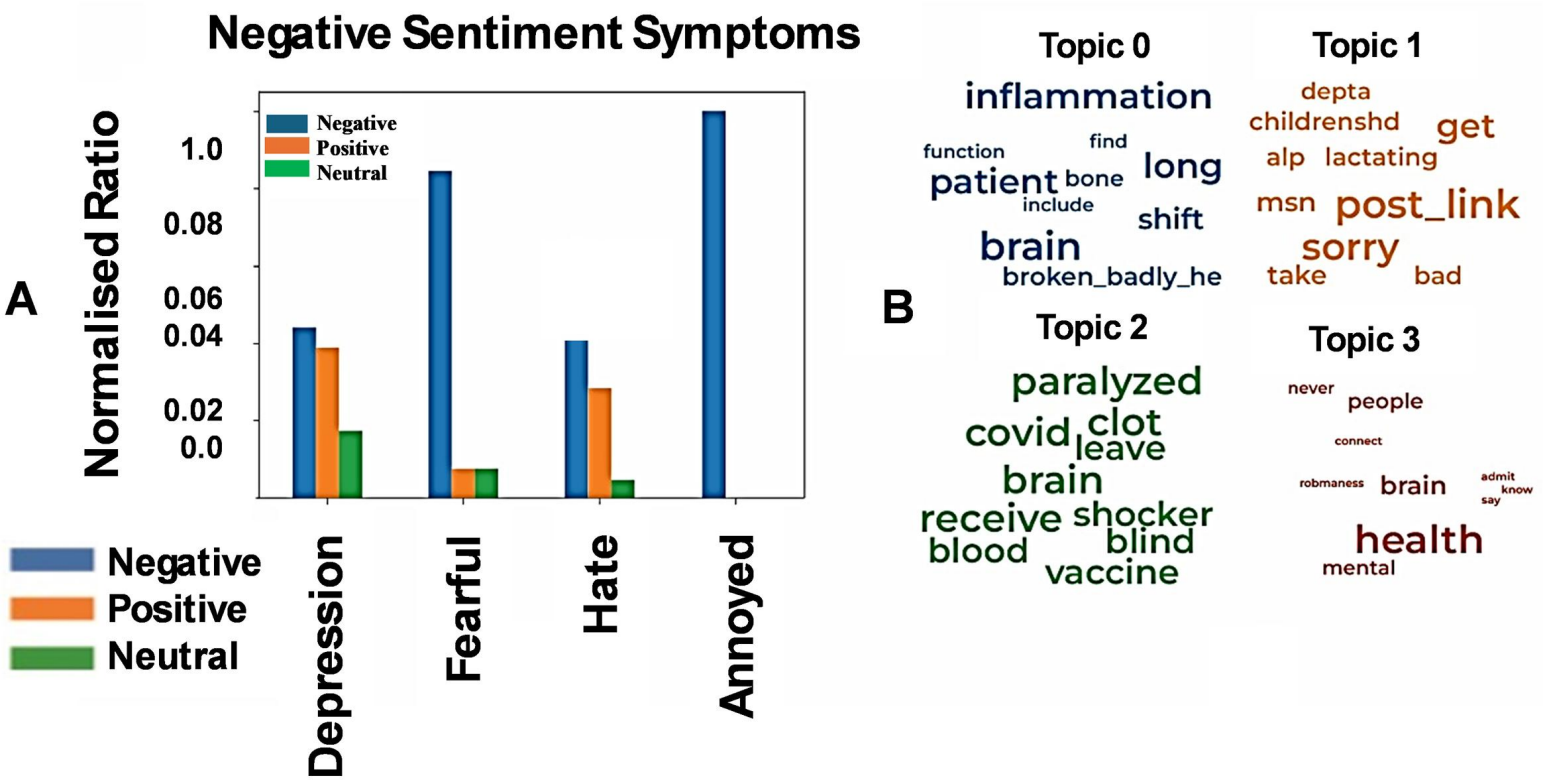
Key themes in negative sentiment X-posts. **(A)** Negative sentiment X-posts frequently conveyed symptoms of anxiety, as expected. Compared to positive sentiment posts, negative sentiment posts included more words reflecting negative moods, with the normalized ratio of negative mood expressions nearing 1. Emotions such as hate, fear, and depression were notably more prevalent in negative sentiment posts. **(B)** The most common topics in negative sentiment X-posts were grouped into four distinct themes based on word frequency, as outlined in the Methods section. The first theme centered on concerns about brain and bone health, along with general worries about inflammation. The second theme focused on concerns about children, particularly infants, with expressions of guilt reflected in words like “sorry” and “bad”. The third theme involved worries about COVID-19, including potential vaccine side effects such as blindness, paralysis, and clotting. The fourth theme addressed mental health, highlighting the need for open discussion, with terms like “admit”, “say”, and “know” commonly used.

We then focused on gaining a deeper understanding of the topics underlying the negative sentiment posts. Our results reflect emotions of feeling down, as evident from the vocabulary used in negative sentiment X-posts (Figure 3A). Upon closer analysis of the topics discussed in these posts, we identified four main themes (Figure 3B). The first theme centers on inflammation and the brain, with terms like “bone” and “broken” possibly hinting at the under-explored brain-bone axis, such as the neural regulation of bone metabolism. The second theme focuses on concerns for children's welfare, reflected in words like “children”, “lactating” ’sorry', and “bad” The third theme addresses ongoing issues related to COVID-19, with terms like “paralyzed”, “clot” and “vaccine,” suggesting concerns about vaccine side effects and their potential connection to worsening COVID-19 symptoms. The fourth theme involves brain health, pointing to a gendered interest in this area of research and possibly reflecting personal struggles, with words like “never”, “admit”, “know”, and ’say' appearing alongside “brain” and “health”. Next, we analyzed the differences in speech acts—such as assertions, questions, and commands—between positive and negative sentiment X-posts. However, no significant differences were found between these two categories in this context.

Cognitive distortions analysis was conducted to better understand the reasons behind the pessimistic approach observed in negative sentiment–identified topics. For the analysis of complaints, we compiled a list of 50 complaint-related words and assessed their frequency across X-posts categorized by sentiment. The ratio of complaint words to total words was highest in negative sentiment X-posts (0.0066), followed by neutral (0.0015) and positive (0.0010) X-posts. A chi-square test of independence confirmed that the distribution of complaint words was significantly associated with sentiment type (*χ*^2^ = 1,407.27, df = 2, *p* < 0.0001). This indicates that negative sentiment X-posts display a stronger tendency to complain about mental health issues (Table 1).

**Table 1:** Cognitive distortions and complaining in health-related X-posts by Sentiment.

Health-Related X-posts	Positive	Negative	Neutral
Blaming	42.9%	44.2%	12.9%
Emotional Reasoning	32.9%	46.6%	20.5%
Global Labelling	37.4%	44.1%	18.4%
Heaven's Reward	21.4%	55%	23.7%
Personalization	42.9%	46.5%	10.7%
Filtering	10.5%	79.5%	10%
Complaining	11%	72.5%	16.5%
Control Fallacy	55.2%	23.9%	20.9%
Control of Fairness	48%	36.7%	15.3%
Fallacy of Change	54.6%	26.5%	18.9%
Polarized Thinking	41.9%	42.6%	15.5%
Mind Reading	41%	17.4%	41.6%
Overgeneration	39.5%	38.4%	22.1%
Ratio of Complaint Words to Total Words by Sentiment Category	11%	72.5%	16.5%

Higher frequencies of cognitive distortions such as "Being Right", "Blaming", "Emotional Reasoning", "Global Labelling", "Heaven's Reward", "Personalization", "Polarized Thinking", "Filtering", and “Complaining” in negative sentiment X-posts. More frequent expressions of distortions like "Control Fallacy" and "Fallacy of Fairness" were detected in positive sentiment X-posts. Results for "Catastrophizing" and "Mind Reading" are inconclusive.

A similar trend was observed in expressions related to cognitive distortions such as “Being Right”, “Blaming”, “Emotional Reasoning,” “Global Labeling,” “Heaven's Reward,” “Personalization”, “Polarized Thinking”, and “Filtering” (Table 1). Interestingly, distortions like “Control Fallacy” and “Fallacy of Fairness” were more frequently expressed in positive sentiment X-posts (Table 1). However, the results for “Catastrophizing” and “Mind Reading” were inconclusive, indicating no significant association with sentiment.

Given the prevalence of negative sentiment and the presence of cognitive distortions in X-posts, we next examined the cognitive load to determine whether the complexity and readability of these posts are related to sentiment and distorted thinking patterns. The cognitive load investigation was based on readability score, sentence count, word count, and average sentence length (Table 1). The readability score, measured by the Flesch-Kincaid Grade Level, was lower in both positive and negative sentiment X-posts compared to neutral ones. Sentence count was largely invariant across posts and was therefore not analyzed further. Both word count and average sentence length differed significantly across sentiment categories (Kruskal–Wallis *H*-tests, *p* < 0.001), with Dunn's *post-hoc* tests revealing that all pairwise comparisons between Negative, Neutral, and Positive posts were highly significant (*p* < 0.001). These results indicate that X-posts with negative or positive sentiment tend to be linguistically simpler and shorter than neutral posts, suggesting that sentiment is strongly associated with variations in linguistic complexity and cognitive load.

**Figure 4:**
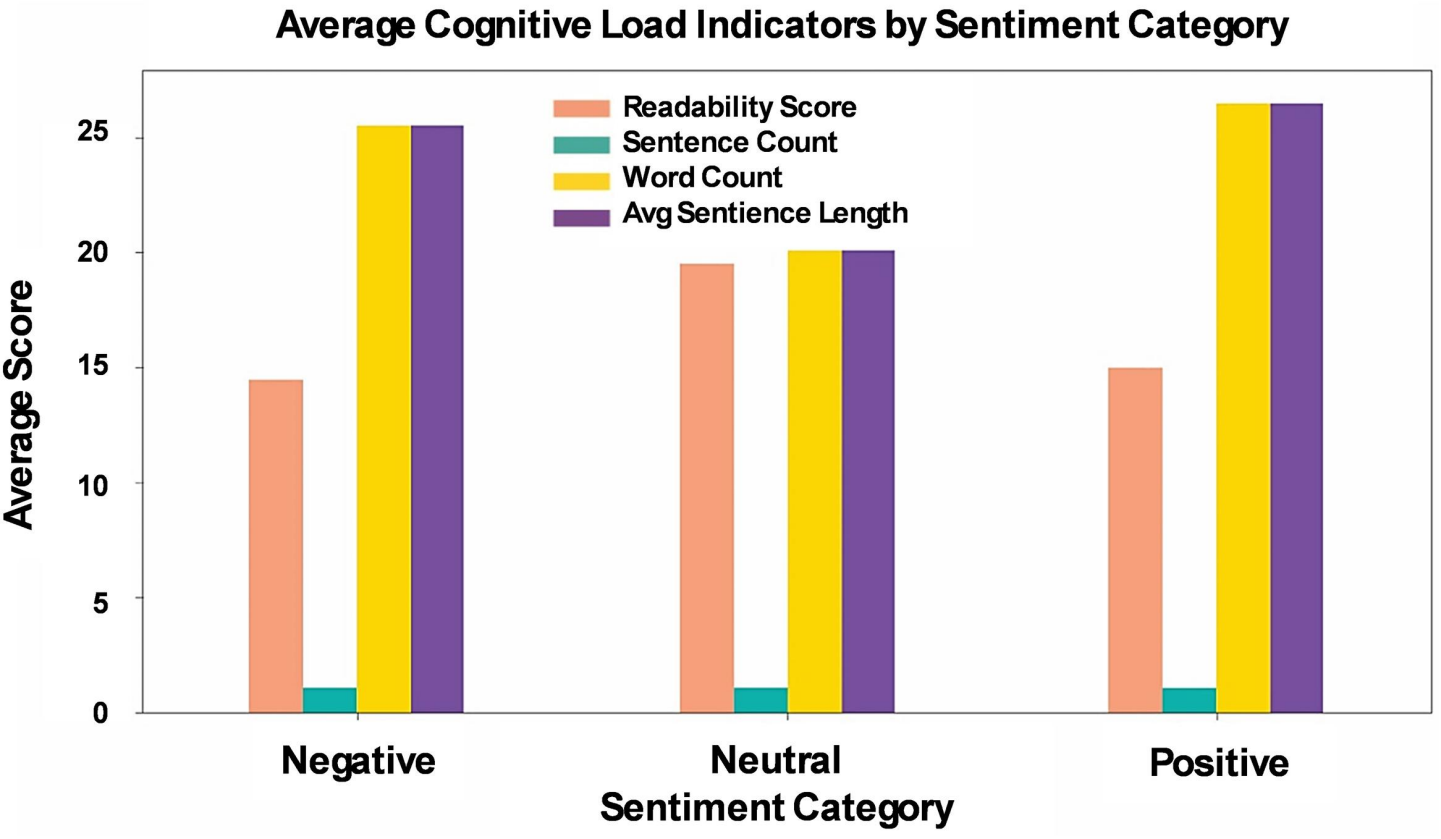
Cognitive load in X-posts: readability, sentence count, word count, and average sentence length across sentiment categories. Word count refers to the total number of words per post, while sentence count is the number of sentences in each post. Average sentence length is calculated as the ratio of word count to sentence count. Readability is assessed using the Flesch-Kincaid Grade Level.

No significant difference in readability was found between positive and negative sentiment posts. However, both categories had lower readability scores on average compared to neutral posts, possibly due to the stronger emotions in positive and negative posts, which may affect sentence clarity. This trend is also reflected in the higher word count for both positive and negative sentiment posts compared to neutral ones. Sentence count did not show significant differences across the sentiment groups (Figure 4).

**Figure 5:**
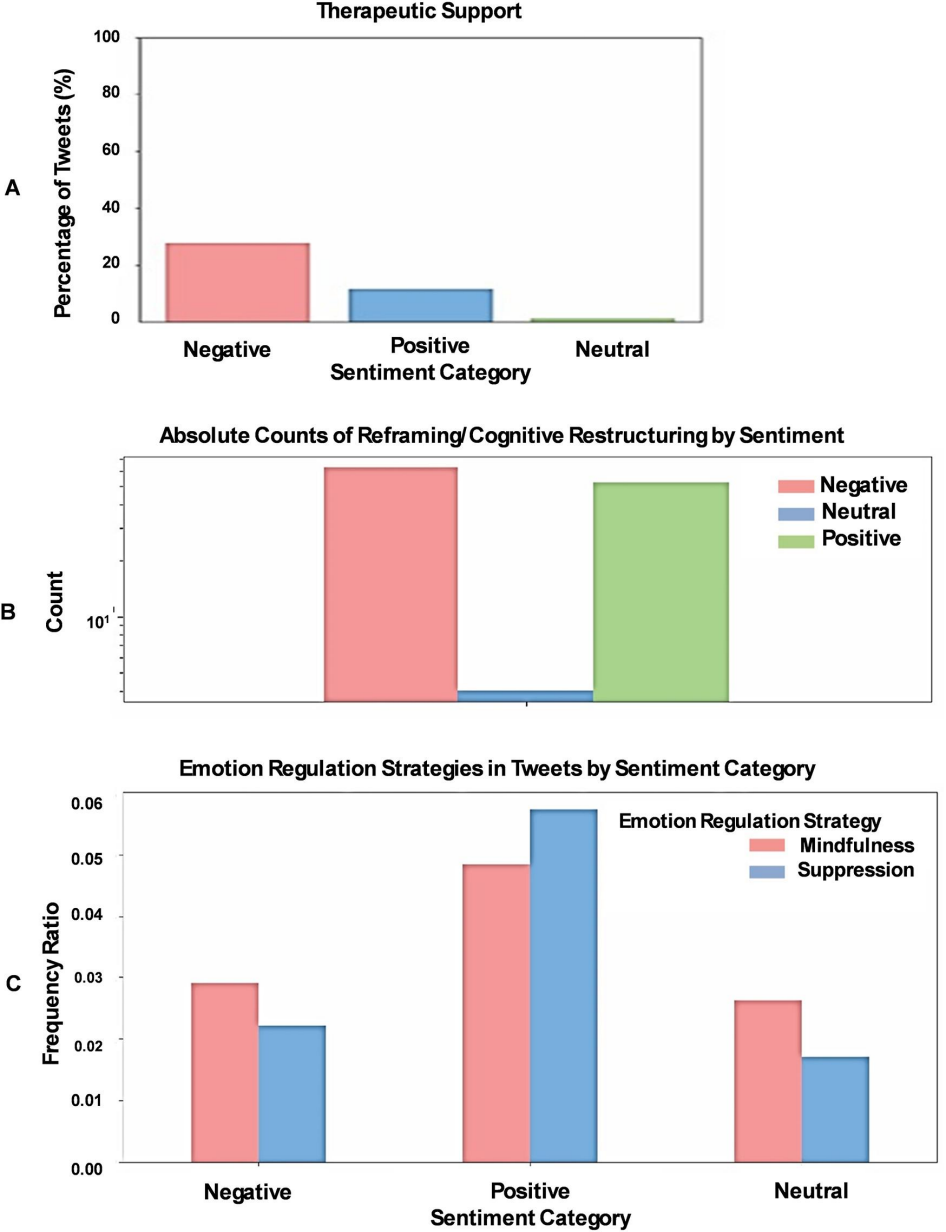
Therapeutic support and cognitive behavioral techniques in X-posts by sentiment. **(A)** Negative sentiment X-posts show a higher frequency of language indicating a desire for therapeutic support compared to positive or neutral sentiment posts. **(B)** Negative sentiment X-posts also display increased use of Cognitive Behavioral Therapy (CBT) techniques, such as reframing and cognitive restructuring. **(C)** Positive sentiment X-posts exhibit a higher prevalence of emotion regulation strategies, such as mindfulness and suppression.

Considering the prevalence of negative sentiment, associated cognitive distortions, and lower readability scores, we next examined how individuals expressing these negative posts attempt to cope with or address these difficulties on a psychological level, focusing on: **(a)** expression of a need for therapeutic support, **(b)** use of Cognitive Behavioral Therapy (CBT) techniques, and **(c)** emotion regulation strategies. Our analysis revealed that negative sentiment X-posts frequently use language indicating a need for therapeutic support, more so than positive or neutral sentiment posts (*χ*^2^ test, *p* < 0.001; Figure 5A). We then investigated the use of CBT techniques, such as reframing and cognitive restructuring (17), and found that negative sentiment X-posts often employ these strategies, suggesting that individuals expressing negative sentiments are more likely to engage in cognitive approaches to shift their perspective (*χ*^2^ test, *p* < 0.01; Figure 5B). Finally, we analyzed X-posts for signs of emotion regulation strategies, including mindfulness and suppression. Our findings indicate that positive sentiment X-posts exhibit notably higher levels of both mindfulness (*χ*^2^ test, *p* < 0.05) and suppression (*χ*^2^ test, *p* < 0.001) compared to negative or neutral posts (Figure 5C).

Extending the analysis of sentiment and psychological coping in X-posts, we next examined how specific food types are associated with sentiment. Across all posts mentioning food, a Chi-square test of independence revealed a highly significant association between sentiment and food type (*χ*^2^ = 29,717.73, *p* < 0.001), indicating that the distribution of food references varied systematically across negative, neutral, and positive posts. For example, wine was more frequently associated with positive sentiment, whereas pizza was disproportionately linked to negative sentiment (Supplementary Information Figure S1A).

We then focused specifically on the subset of posts that discussed stress management. Within this context, food mentions again showed a significant association with sentiment (*χ*^2^ = 621.59, *p* < 0.001). Positive sentiment stress-related posts most frequently mentioned wine, yogurt, and bread, whereas negative sentiment stress-related posts often referenced pizza, onion, and nuts (Supplementary Information Figure S1B). Together, these results demonstrate that food references are not sentiment-neutral but instead reflect distinct affective and coping contexts.

Following our examination of sentiment in relation to foods and stress management, we next compared drug, and supplement mentions across different sentiment types. A Chi-square test of independence revealed a significant association between drug mentions and sentiment categories (Chi-square = 3,366.75, df = 56, *p* < 0.001), indicating that certain drugs were disproportionately mentioned in specific sentiment contexts. Negative sentiment was linked to drugs such as lorazepam, sodium fluoride, and simvastatin, whereas positive sentiment was associated with medications including doxycycline, pregabalin, famotidine, haloperidol, guaifenesin, clozapine, titanium dioxide, and hydrocortisone (Supplementary Information Figure S2A). These results suggest a clear differentiation in how drugs are discussed across sentiment types. Interestingly, a similar pattern was observed for dietary supplements. A Chi-square test for supplement mentions confirmed a significant association with sentiment categories (Chi-square = 94.0, df = 44, *p* < 0.001), indicating that certain supplements were disproportionately mentioned in specific sentiment contexts. Silymarin, beetroot, wheatgrass, and chaga mushroom were predominantly associated with positive sentiment, while red clover, green coffee bean extract, valerian root, and hawthorn berry were primarily associated with negative sentiment (Supplementary Information Figure S2B). These findings highlight that both pharmaceutical and nutraceutical discussions on social media are closely linked to sentiment, reflecting distinct patterns of public perception and engagement.

## Discussion

The analysis of X-posts shows that the sentiment expressed within these X-posts offers a window into how individuals perceive the world around them. The analysis of X-posts reveals how sentiment reflects individuals' perceptions of their environment. Positive sentiment X-posts are often characterized by optimism and a proactive approach to health issues. This is consistent with findings from recent studies that suggest individuals who exhibit positive emotions are more likely to focus on personal growth and recovery. For instance, the frequent use of terms such as “health benefit,” “brain health,” “improve,” and “learn” in positive X-posts aligns with the concept of positive reframing, which emphasizes viewing challenges as opportunities for improvement (Figure 2A) (30, 31). Conversely, negative sentiment X-posts frequently convey a more apprehensive or distressed perspective (Figure 2B & Figure 3). Terms like “ paralyzed,” “health shocker,” and “COVID” highlight significant health concerns and adverse events (Figure 3B). This aligns with research showing that negative emotions are often associated with a heightened focus on threats and risks (32). These findings underscore how emotional states influence health perceptions: positivity is often linked to resilience and a growth-oriented mindset, while negativity is associated with heightened fear and apprehension. This distinction highlights the importance of emotional context in shaping how individuals interpret and react to health-related information.

The prevalence of cognitive distortions in X-posts reveals underlying emotional states, with negative sentiment posts often showing signs of depression, while positive sentiment posts reflect more optimistic cognitive patterns. To further understand how sentiment shapes individuals' perceptions and reactions, we examined the cognitive distortions present in X-posts and their association with positive and negative emotional states. The overrepresentation of various cognitive distortions with negative sentiment may indicate depressive disorders. X-posts exhibit multiple known cognitive distortions (Table 1). One common distortion in posts expressing negative sentiment is blaming, with 44% of statements involving blame linked to negative moods. These posts often reflect the struggle to identify the cause of negative feelings (33, 34). In some cases, blame is directed inward, manifesting as self-blame. A notable example from X-posts classified as negative sentiment and exhibiting a blaming cognitive distortion reads: “*I am so sad, my bad mental health over the past few years caused me to lose so much YouTube momentum. I miss how engaged the community used to be over there, and I know a lot of it's my fault. I hate my brain, but I am going to make some gold on there again eventually*”. In this post, blame is directed toward the user, potentially indicating depressive symptoms (35, 36). Global labeling, another cognitive distortion, is also overrepresented in negative sentiment posts (44%), further highlighting symptoms of depression. For example, a user wrote: “*All the health books are like: do these things you hate, eat these things you don't like, otherwise you’ll deteriorate and die*.” Using words such as “*all*,” “*hate*” and “*die*” reflects global labeling distortions and hints at suffering from depressive symptoms. Personalization, which involves attributing health problems or negative outcomes to oneself and increasing feelings of guilt and inadequacy, was also overrepresented, with over 46% of negative sentiment posts expressing personalization (37). Lastly, filtering is evident in the focus on negative aspects of health-related issues while ignoring any positive elements, reinforcing a negative perception (38). Filtering was associated with negative sentiment in more than 79% of the cases. These findings align with Beck's cognitive theory of depression, which suggests depressed people have negative and hopeless thoughts or core beliefs about themselves, the world, and the future (39). In contrast, positive sentiment X-posts are more commonly associated with cognitive distortions like “Control Fallacy” and “Fallacy of Fairness.” While these distortions still reflect deviations from reality, they suggest a different cognitive focus compared to those observed in negative sentiment X-posts. According to Carver and Scheier, individuals with positive outlooks may struggle with unrealistic expectations, such as the belief in excessive personal control or fairness, but they generally maintain a more optimistic perspective (40). This is consistent with Dweck's research on mindsets, which indicates that positive sentiment is often linked to adaptive coping strategies and a resilient cognitive style, despite the presence of certain distortions (41).

The way individuals cope with stress and health concerns also varies depending on whether their X-posts express positive or negative sentiments. Extending from the observed cognitive distortions, we explored how these sentiment-linked thought patterns translate into different coping strategies for managing stress and health concerns. We found that Negative sentiment X-posts are more likely to include language indicative of a need for therapeutic support, as well as references to Cognitive Behavioral Therapy techniques such as reframing and cognitive restructuring (Figure 5). This suggests that individuals expressing negative sentiment may be actively trying to manage their distress through cognitive strategies aimed at changing their perspective or thought patterns. In contrast, positive sentiment X-posts tend to reflect coping mechanisms like mindfulness and suppression, which are strategies that help individuals maintain a positive outlook despite challenges (42). This difference in coping styles indicates that individuals with positive sentiments might be better equipped to manage stress and maintain emotional balance, while those with negative sentiments are more focused on trying to alter their cognitive and emotional responses to stress.

Additionally, the types of foods and drugs mentioned in relation to various sentiments provide further insights into coping behaviors. Positive sentiment X-posts are associated with foods such as wine, yogurt, and bread, which are linked to stress management, as well as drugs and supplements such as doxycycline, pregabalin, and Chaga mushrooms. Interestingly, doxycycline has been shown to protect against neurodegenerative diseases by inhibiting *α*-synuclein-associated pathologies and reducing neural toxicity (43, 44). Pregabalin appears to be effective in alleviating pain (45). Chaga mushrooms are known for their anti-cancer properties; however, they can also induce serious side effects in the renal system (46, 47). Conversely, negative sentiment X-posts more frequently mention comfort foods like pizza, along with substances such as lorazepam and valerian root, suggesting a reliance on both food and medication to manage stress and anxiety (48). However, consuming a carbohydrate-rich diet, such as pizza, can lead to extreme obesity (48, 49). Additionally, valerian root has been reported to potentially cause encephalopathy. These choices reflect a focus on health and well-being in both positive and negative X-posts, but they also highlight the dangers of conveying inappropriate health-related messages through X and other social media platforms.

The research on X posts encounters several limitations due to selection bias. Self-selection bias occurs because individuals who are particularly concerned about brain health or hold strong opinions may be more inclined to post, skewing the data toward more extreme sentiments and not accurately reflecting the broader population's views (50). Additionally, platform bias is evident, as the nature of X amplifies negative or sensational content, which is more frequently shared or reposted, thus distorting sentiment analysis (51). This bias is further compounded by the brief and oversimplified nature of X posts; the 280-character limit can result in incomplete or distorted expressions of complex emotions. Temporal bias also affects the results, as the time frame of data collection can influence sentiment based on recent events or health crises, impacting the generalizability of the findings.

The analysis of negative sentiment in X posts regarding brain health reveals critical areas for public health communication and policy. The high prevalence of distressing emotions such as anxiety and fear highlights the need for empathetic and clear public health messaging that addresses specific concerns, including inflammation and COVID-19, and their impact on mental health (52). To improve mental health outcomes, interventions should target cognitive distortions commonly found in negative posts, promote positive coping strategies like mindfulness, and ensure accessible therapeutic support (53). Additionally, public health policies must address misinformation about dietary supplements and medications while integrating insights from social media to refine communication strategies and respond proactively to emerging public health concerns.

## Conclusion and recommendations

The main conclusion of the text is that sentiment analysis of Twitter X posts related to brain health reveals distinct emotional patterns and coping strategies that have significant implications for public health communication and policy. The study finds that negative sentiments are prevalent and often linked to severe health concerns, including brain inflammation and COVID-19, and are associated with higher levels of cognitive distortions and complaints. In contrast, positive sentiments emphasize resilience and proactive health management. These insights highlight the necessity for targeted public health messaging, informed by social media trends, to address specific health anxieties, correct misinformation about dietary supplements and medications, and foster effective coping mechanisms.

## Data Availability

The data that support the findings of this study are available from the corresponding author upon reasonable request.
