# Supplementary material for: Dissecting the difference between positive and negative brain health sentiment using X data
Source: Front Digit Health. 2025 Nov 26;7:1648671. doi: 10.3389/fdgth.2025.1648671 (PMC12690490; doi:10.3389/fdgth.2025.1648671)
Supplement: Supplementary file 1 [file Datasheet1.pdf]

Dissecting the Difference Between Positive and Negative Brain Health Sentiment Using X Data

Author information & Affiliations

Piotr Religa[1,*], **Michel-Edwar Mickael**[2,*], **Marzena Lazarczyk**[2], **Norwin Kubick**[3], **Ibrahim F. Rehan**[2] **Jarosław Olav Horbańczuk**[2], **Asmaaa Elnagar**[2], **Mariusz Sacharczuk**[2] and **Atanas G. Atanasov**[1,3,4,*]

[1] Department of Medicine, Karolinska Institute, Visionsgatan, Solna, Sweden.
[2] Institute of Genetics and Animal Biotechnology of the Polish Academy of Sciences, Jastrzebiec Magdalenka, Poland, (ML, JOH, AE, MS).
[3] Department of Biology, Institute of Plant Science and Microbiology, University of Hamburg, Ohnhorststr, Hamburg, Germany, (NK).
[4] Ludwig Boltzmann Institute of Digital Health and Patient Safety, Medical University of Vienna, Spitalgasse, Vienna, Austria.

[*] **Correspondences**: **Atanas G. Atanasov** (AGA), email: a.atanasov.mailbox@gmail.com; **Michel-Edwar Mickael** (M-EM), email: m.mickael@igbzpan.pl; and **Piotr Religa** (PR), email: piotr.religa@ki.se. PR and M-EM contributed equally to the manuscript.

Supplementary Information

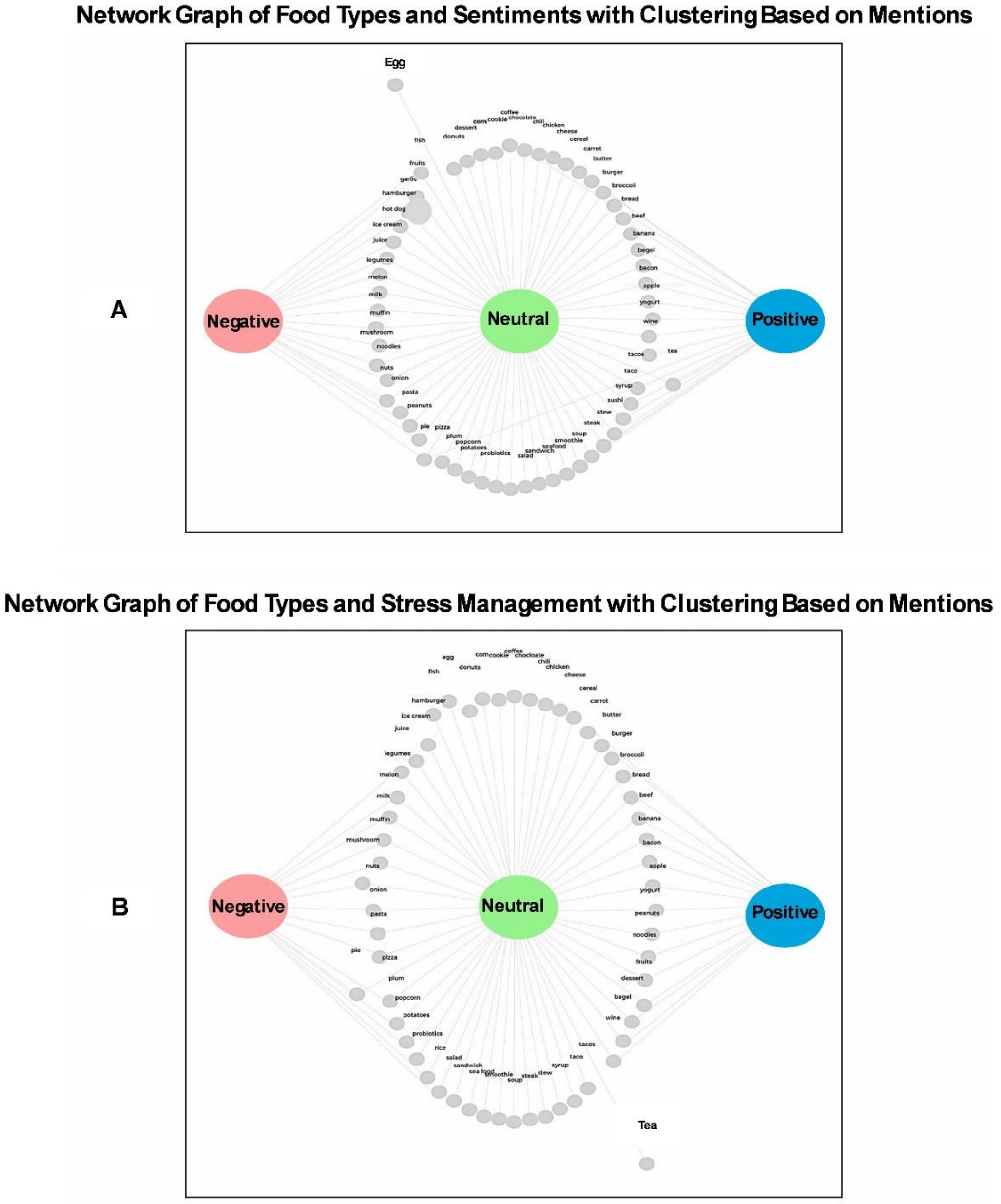

FIGURE S1 | Relationship Between Food Types and X (formerly Twitter) Sentiment, and Associations with Stress Management. We categorized dietary and food options based on their frequency of appearance in each sentiment group, as outlined in the methods section. **(A)** Certain food types are associated with negative sentiments, including fish, garlic, hamburgers, hot dogs, ice cream, juice, legumes, onions, peanuts, pie, and pizza. The association with these foods may indicate a desire for healthier choices, such as fruits, fish, and nuts, or a tendency to manage negative emotions by opting for less healthy options like hamburgers, ice cream, and pizza. Conversely, positive sentiment posts are more frequently linked to foods and drinks that are generally perceived positively, such as wine, tea, apples, and bananas. **(B)** A deeper analysis explored whether these food choices are associated with stress management. We found that

foods linked to stress management within the negative sentiment category included a polarized range of options: less healthy choices like pizza, hamburgers, ice cream, muffins, and pies, alongside healthier options like legumes. In contrast, positive sentiment posts addressing stress management consistently featured mood-enhancing options like wine, tea, various fruits, and peanuts.

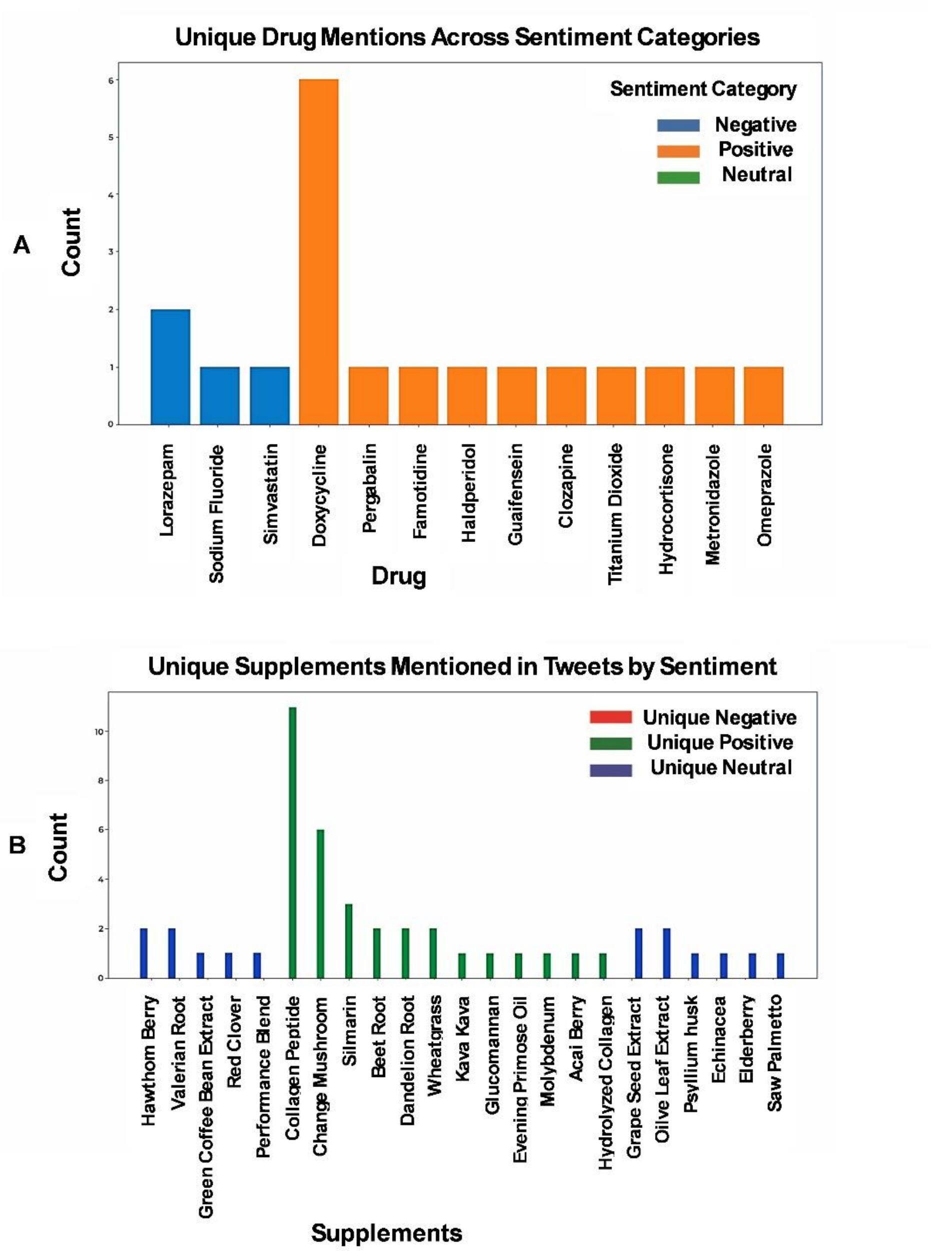

FIGURE S2 | Top Drugs and Drug Supplements Mentioned in X-Posts Classified by Sentiment Type. **(A)** We measured the frequency of drug mentions in relation to each sentiment category. Certain drugs, particularly doxycycline, were more frequently mentioned in positive sentiment posts, while others, such as sodium fluoride, were more commonly associated with negative sentiment. **(B)** We also investigated the use of supplements by counting mentions across the three sentiment categories. Our analysis revealed a higher frequency of certain supplements linked to positive sentiment, including collagen peptides and chaga mushrooms.
